# Infiltrating ductal carcinoma breast, metastatic to ipsilateral axillary lymph nodes harbouring primary tuberculous lymphadenitis: a case report

**DOI:** 10.11604/pamj.2014.18.167.1494

**Published:** 2014-06-19

**Authors:** Pinki Pandey, Alok Dixit, Aparna Tanwar, NC Mahajan

**Affiliations:** 1 Departments of Pathology, M M Institute of Medical Sciences and Research, Mullana, Ambala. 133203. Haryana, India; 2 Departments of Pharmacology, M M Institute of Medical Sciences and Research, Mullana, Ambala. 133203. Haryana, India

**Keywords:** Ductal carcinoma, breast, lymph node, tuberculous

## Abstract

The coexistence of breast cancer and tuberculosis has been described in over 100 cases; however its coexistence in the axillary lymph node is rare with only a handful cases have been reported in the literature. We report a case of infiltrating ductal carcinoma of the left breast, metastatic to ipsilateral axillary lymph nodes harbouring tuberculous lymphadenitis without primary mammary or pulmonary tuberculosis. The case is presented for its rarity and illustrates that the simultaneous occurrence of tuberculosis and carcinoma can create a dilemma in the diagnosis and treatment, so surgeons and pathologists should keep such a combination on the back of their mind, especially in endemic areas.

## Introduction

The simultaneous occurrence of tuberculosis and carcinoma is unusual, creating a dilemma in the diagnosis and treatment and association of the two has baffled surgeons and physicians for over two centuries. The coexistence of breast cancer and tuberculosis has been described in over 100 cases, [1–4] however the coexistence of metastatic breast cancer in primary tubercular lymph nodes is extremely rare with only a handful cases have been reported in the literature. [5–9] We report a case of infiltrating ductal carcinoma of the left breast, metastatic to ipsilateral axillary lymph nodes harbouring tuberculous lymphadenitis wherein no evidence of tuberculosis was found elsewhere.

## Patient and observation

A 50 year old lady presented with the history of a lump in the left breast for one year duration. The lump was painless and slowly increasing in size. There was no family history of breast cancer or any history of taking oral contraceptives or estrogen therapy. There was no history of nipple discharge. Physical examination was unremarkable. The rest of the systemic examination revealed no abnormality. Local examination of the left breast showed 6.0x3.0 cm large, hard, irregular, non- tender mobile lump involving mainly the lower outer quadrant. There was puckering of the overlying skin with retracted nipple. Two lymph nodes were palpated in her left axilla and both were slightly tender. Contralateral breast and axilla were normal. The patient was referred for fine needle aspiration cytology (FNAC) with the clinical diagnosis of carcinoma of the left breast with axillary lymph nodes involvement. FNAC from the breast mass showed clusters, acinar pattern and dissociative population of pleomorphic cells having large nuclei with irregular nuclear membrane, anisonucleosis, prominent nucleoli and moderate amount of cytoplasm. FNAC from the clinically significant axillary lymph nodes showed the presence of similar sheets and groups of malignant tumor cells against clear lymphoid background. Diagnosis offered on cytology was ductal carcinoma of the left breast with metastasis to the left axillary lymph nodes. The preoperative workup consisted of a complete haemogram, ESR, chest X- ray and ultrasound examination of the abdomen. Complete haemogram showed mild microcytic hypochromic anemia while other investigations were within normal limits. The patient subsequently underwent modified radical mastectomy of the left breast with axillary lymph nodes clearance and the specimen was sent for histopathology examination.

### Pathological examination

The mastectomy specimen measured 16x15x4 cm and the axillary tail was 8.0 cm in length. The overlying skin showed puckering whereas nipple was retracted. On slicing, the specimen revealed an irregular yellowish gray mass measuring 3.5x2.5x1.5cm in size. It was firm in consistency with infiltrating margins. On sectioning there was a gritty sensation. The axillary tail revealed 14 lymph nodes ranging from 0.5cm to 1.0cm in diameter, and all were sampled.

**Figure 1:**
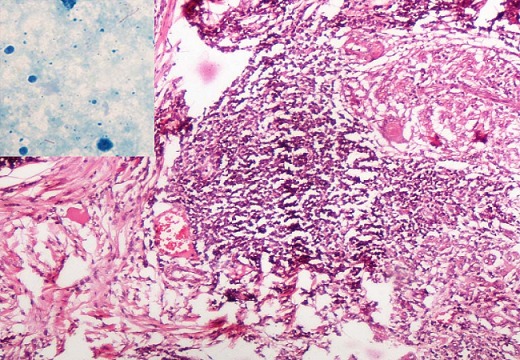
Microphotograph of lymph node revealing multiple well formed caseating granulomas composed of epithelioid cells, Langhans’ giant cells and metastatic invasive ductal carcinoma (H&E, X100). Inset shows acid-fast bacilli (Ziehl- Neelsen stain, oil immersion)

**Figure 2:**
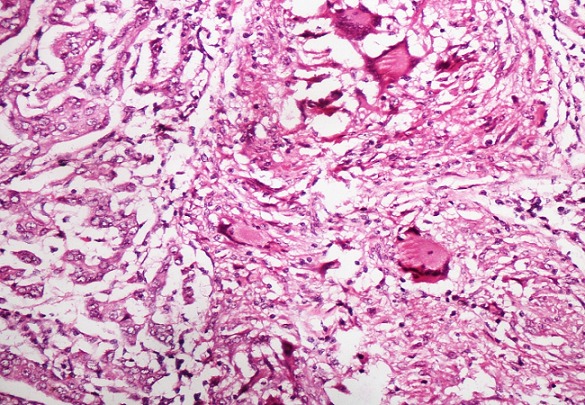
High power view of the lesion showing granulomatous foci composed of epithelioid cells, Langhans’ giant cells and metastatic invasive ductal carcinoma in the same field. (H&E, X200)

Representative samples were fixed in 10% neutral buffered formalin, dehydrated and embedded in paraffin. Sections were stained in haematoxylin and eosin. Microscopic examination of the tumor showed sheets, clusters and duct- like arrangement of tumor cells of having large vesicular nucleus containing prominent nucleoli embedded within dense collagenous stroma. Of the fourteen lymph nodes, thirteen showed tuberculous lymphadenitis in the form of numerous well formed caseating granulomas composed of epithelioid cells, Langhans’ giant cells surrounded by lymphocytes, two of the lymph nodes showed coexistent carcinomatous deposits along with well defined caseating granulomas and reactive hyperplasia in one. Ziehl Neelsen stain for acid- fast bacilli was positive (Figure 1, Figure 2) Sections from resected margins, deep resection plane and nipple and areola did not show any evidence of infiltration by tumor. The surrounding breast was normal. The final diagnosis offered on histopathology was infiltrating ductal carcinoma, grade II (Nottingham modification of Scarff Bloom Richardson System) of the left breast with metastasis in left axillary lymph nodes harbouring tuberculous lymphadenitis.

Since preoperatively tuberculosis was not suspected, Mantoux test, culture, serology or polymerase chain reaction were not performed. On the basis of histopathological report, the patient was put on anti- tuberculous therapy along with adjuvant chemotherapy with cyclophosphamide, methotrexate and 5- fluorouracil (CMF) and responded well to treatment.

## Discussion

Tuberculosis remains a major threat to global health, an estimated 8,000,000 new cases and 3,000,000 deaths annually worldwide. [10] Tuberculous lymphadenitis is the commonest form of extra pulmonary tuberculosis with involvement of cervical, axillary and inguinal lymph nodes. In children axillary tuberculous lymphadenitis secondary to BCG vaccination or pulmonary and cutaneous tuberculosis is not uncommon, but primary or isolated axillary lymph node involvement in adults without clinical evidence of any other organ or systemic involvement is extremely rare. When it is impossible to pinpoint any source of infection, the only possible explanation of tuberculosis limited to lymph nodes could be either a retrograde spread from the mediastinal nodes, or hematogenous spread from a sub clinical focus which was not picked up by the routine investigation.

The synchronous occurrence of tuberculosis and cancer existing in the same patient was first described by Warthin in 1899 and later by many authors in many diverse ways. [1] The coexistence of breast cancer and tuberculosis has been described in over 100 cases, [1–4] however its coexistence in the axillary lymph node is rare. Das et al7 reported a case of colloid carcinoma of the breast while Pandey et al 8 reported infiltrating ductal carcinoma metastatic to axillary lymph nodes containing tuberculous foci. There was no evidence of tuberculosis elsewhere in these patients. Kaplan et al reviewed 58,245 patients with cancer and identified 201 cases of coexisting tuberculosis. [2] Among 14,742 cases of breast reviewed by coexistence was identified in their series. In the present case, of the fourteen axillary lymph nodes isolated, a plethora of pathological findings ranging from reactive hyperplasia, tuberculosis and a combination of tuberculosis and metastasis were noticed.

Close association of two pathological lesions always incite a debate about their etiological relationship. Coexistence of tuberculosis and carcinoma may be a simple co- incidence because of high incidence of tuberculosis in India or it can be proposed that one disease process might lead to the other.

In our case, FNAC from the breast and axillary lymph nodes revealed infiltrating ductal carcinoma with metastasis in axillary lymph nodes. FNAC failed to detect mixed lesions from axillary lymph nodes. Therefore multiple punctures from many sites should be performed. The accurate diagnosis was only achieved after histopathological examination.

## Conclusion

The present case is a coincidental but a noteworthy example which illustrates that surgeons and pathologists should keep such a combination on the back of their mind, especially in endemic areas. The simultaneous occurrence of tuberculosis and carcinoma can create a dilemma in the diagnosis and treatment.
